# Peripheral and cognitive benefits of physical exercise in a mouse model of midlife metabolic syndrome

**DOI:** 10.1038/s41598-022-07252-x

**Published:** 2022-02-28

**Authors:** Farida El Gaamouch, Hsiao-yun Lin, Qian Wang, Wei Zhao, Jiangping Pan, Kalena Liu, Jean Wong, Clark Wu, Chongzhen Yuan, Haoxiang Cheng, Weiping Qin, Ke Hao, Bin Zhang, Jun Wang

**Affiliations:** 1 Department of Neurology, Icahn School of Medicine at Mount Sinai, 1 Gustave L. levy Place, New York, NY 10029 USA; 2 Department of Genetics and Genomic Sciences, Icahn School of Medicine at Mount Sinai, New York, NY 10029 USA; 3 Mount Sinai Center for Transformative Disease Modeling, Icahn School of Medicine at Mount Sinai, New York, NY 10029 USA; 4 Icahn Institute of Genomics and Multi-scale Biology, Icahn School of Medicine at Mount Sinai, New York, NY 10029 USA; 5 James J Peters VA Medical Center, Research & Development, Bronx, NY 10468 USA

**Keywords:** Diseases, Nutrition disorders, Obesity, Neuroscience, Learning and memory, Fear conditioning

## Abstract

Despite national and international efforts for the prevention of metabolic syndrome and its underlying diseases/disorders, its prevalence is still rising, especially in the middle-aged population. In this study, we explore the effect of high fat diet on the development of metabolic syndrome in middle-aged mice and to evaluate the potential benefits of voluntary physical exercise on the periphery as well as brain cognitive function, and to explore the potential mechanisms. We found that metabolic syndrome developed at middle age significantly impairs cognitive function and the impairment is associated with gene dysregulation in metabolic pathways that are largely affecting astrocytes in the brain. Eight-week voluntary wheel running at a frequency of three times a week, not only improves peripheral glucose control but also significantly improves learning and memory. The improvement of cognitive function is associated with restoration of gene expression involved in energy metabolism in the brain. Our study suggests that voluntary physical exercise is beneficial for metabolic syndrome-induced peripheral as well as cognitive dysfunction and can be recommended as therapeutic intervention for metabolic syndrome and associated diseases.

## Introduction

Metabolic syndrome (MetS) is defined as having at least three out of five cardio-metabolic conditions that include abdominal obesity, hyperglycemia and impaired insulin sensitivity, hypertriglyceridemia, hypercholesterolemia and hypertension^1,2^. It is by far the most prevalent disease in the world. Based on the National Health and Nutrition Examination Survey^3^, over 30% of adults aged 18 years or older in the United States are estimated to have MetS. In Europe and Latin America, approximately 25% of the adult population meet the criteria for metabolic syndrome^4^. It is projected that the prevalence of MetS will continue to increase worldwide because of the rising rate of obesity and diabetes in developing countries^5,6^.

MetS is associated with increased risk of multiple chronic illnesses including cardiovascular disease^7–9^, chronic kidney disease^10,11^ and several types of cancers such as liver, colorectal and bladder cancer^12^. Systemic review and Meta-analysis of clinical and population-based studies showed that metabolic syndrome is also associated with increased risk of vascular dementia and increased risk of progression from mild cognitive impairment to dementia^13^. Several longitudinal studies also demonstrated that metabolic syndrome, as a whole, is related to a higher risk of cognitive decline^14–16^. Based on these epidemiological data coupled together with an aging population in many Western countries, the potential impact of metabolic syndrome has serious implications on general health and on the health care system. Therefore, safe and efficacious interventions for metabolic syndrome and associated peripheral and central disorders are of fundamental importance.

While the etiology of MetS is poorly understood it is generally acknowledged that both genetic and environmental factors play roles in the development of MetS. Genetic susceptibility to MetS has been under active investigation^17^ and select mutations have been linked to the key components of MetS^18^. For example, Polymorphisms of *APM1, a* gene coding for adipocyte-derived hormone Adiponectin, are significantly associated with obesity in Hispanic population^19^. Genetic link between the angiotensionogen gene (*AGT*) and essential hypertension was also reported^20,21^. Apolipoprotein E and C-III (*APOE* and *APOC3*) are linked to plasma levels of cholesterol and triglycerides^22,23^. Age is also considered as a risk factor for MetS. The prevalence of MetS increases with age. In males and females under the age of 40, about 16–20% in the U. S. meet the criteria for MetS while 41% males and 37% females age between 40 and 59 meet the same criteria. Over 50% males and females 60 years of age and older meet MetS criteria^24^. This is not surprising because individual risk factors to MetS increase with age^25^. The other important environmental factor is lifestyle, which includes dietary composition and daily activity. For example, prospective and clinical studies demonstrated that the Mediterranean diet was associated with lower prevalence of MetS and adherence to the Mediterranean diet benefits components of MetS, including blood pressure, waist circumference, plasma triglycerides and lipoprotein cholesterol, and glucose^26,27^. A sedentary life style is associated with MetS. Meta-analysis showed that people spending more time in sedentary behaviors have greater odds of having MetS^28^. Accumulating evidence suggests that moderate physical exercise (PE) has broad health benefits on individual risk components to MetS such as obesity, hypertriglyceridemia and low HDL cholesterol ^29,30^ and moderate-to-vigorous physical activity may prevent MetS^28^.

Preclinical studies also showed that high fat diet can induce features of MetS in rodent models. C57BL/6 mice fed on a high fat diet develop obesity, hyperglycemia, dyslipidemia and impaired glucose tolerance^31–33^ and have been used as a model to study various aspects of MetS. Because of the high prevalence of MetS in middle and advanced age, in this study we initiated the diet at 12 months of age, equivalent to middle-age in humans. We also focused on females as majority of the animal studies have been conducted on males, and there is a clear sex-dimorphism between men and women^34^. Though the prevalence of MetS is similar between men and women, the pathophysiology and its contributory factors are different. For example, in men, the main contributory factors are hypertension and high levels of triglycerides while in women, increased BMI and waist circumference, hyperglycemia and low HDL are the major contributors^35^. In this study, we tested the effect of MetS developed in middle-aged female mice and the potential benefits of voluntary physical exercise (PE) on peripheral as well as brain dysfunction induced by MetS. Our studies may provide a basis for the development of safe and effective means to modulate MetS-mediated complications in the brain and the periphery.

## Results

### High fat diet induces features of metabolic syndrome

**Figure 1:**
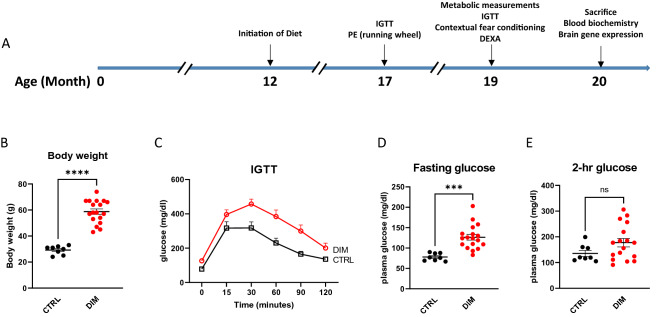
Scheme of the experimental design and the effect of high fat diet in middle-aged female mice (**A**) Scheme of the experimental design. (**B**–**E**) Five-month high fat diet on body weight and glucose utilization as reflected by (**C**) IGTT, (**D**) fasting glucose and (**E**) post prandial glucose in plasma. Data represents mean ± SEM, n = 8–18 per group.

In this study, we initiated the high fat diet treatment when the C57BL/6 mice reached 12 months of age (Fig. 1A for schematic design of the study). This age in mice recapitulates the physilogical conditions equivalent to middle-age in humans. Following 5 months of high fat diet treatment, the DIM mice became obese and had significantly higher body weight compared to the CTRL mice on 10% fat diet (Fig. 1B, two-tailed t-test, t_24_ = 9.090, P < 0.0001). The DIM mice developed impaired IGTT compared to the CTRL mice (Fig. 1C, two-way ANOVA repeated measure, F_1,24_ = 8.616, P = 0.0072 for diet effect; F_5,120_ = 1.695, P = 0.1410 for interaction). The DIM mice had significantly increased fasting glucose content (Fig. 1D, two-tailed t-test, t_24_ = 4.478, P = 0.0002). There were no significant differences in two-hour post-prandial glucose content (Fig. 1E, two-tailed t-test, t_24_ = 1.508, P = 0.145). We then randomly group the DIM mice to DIM-CTRL and DIM-PE (Body weight 58.2 ± 10.0 g vs. 59.3 ± 8.0 g; fasting glucose 124.7 ± 34.8 mg/dl vs. 128.2 ± 25.5 mg/dl) where both groups of mice were continually fed on the high fat diet while the DIM-PE mice were subjected to voluntary running wheel exercise three times a week for 8 weeks (Fig. 1A).

### Benefits of PE on glucose utilization and body composition

**Figure 2:**
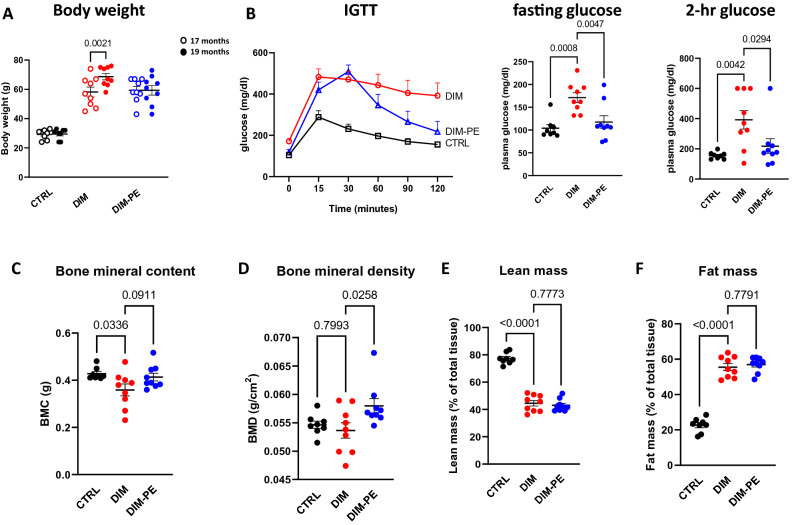
The effect of voluntary wheel-running on periphery glucose and bone health measurements of (**A**) Body weight, (**B**) glucose utilization: IGTT, fasting glucose and postprandial glucose in plasma, (**C**–**D**) Bone mineral content and bone mineral density, (**E**–**F**) Lean and fat mass following two months voluntary physical exercise. Data represents mean ± SEM, n = 8–9 per group.

The DIM-PE mice were voluntarily exercising on the running wheel three days a week for 8 weeks. We calculated that the mean exercise intensity is about 4.1 km over 24 h period with large variations between animals (ranging from 0.47 km to 13.0 km). Following 2 months running wheel exercise, the DIM-PE mice had stabilized their body weight (Fig. 2A, two-tailed t-test, t_8_ = 0.000, P > 0.999) while the DIM-CTRL mice continued to gain body weight (Fig. 2A, two-tailed t-test, t_8_ = 4.452, P = 0.0021). IGTT test also showed that the DIM-CTRL mice continue to have impaired glucose tolerance (Fig. 2B, left panel, two-way ANOVA repeated measure, F_2,23_ = 14.06, P = 0.0001 for diet/PE effect; F_,10,115_ = 3.112, P = 0.0015 for interaction) with higher fasting glucose (Fig. 2B, middle panel, One-way ANOVA, F_2,23_ = 9.903, P = 0.0008) and higher two-hour post-prandial glucose content (Fig. 2B, right panel, One-way ANOVA, F_2,23_ = 6.595, P = 0.0054) while DIM-PE mice had similar fasting glucose level as well as two-hour post prandial glucose level as the LF-CTRL mice (Fig. 2B), indicating the benefit of physical activity on better controls of peripheral glucose level.

We then subjected all mice to DEXA scan to evaluate body composition. We found that there were differences in bone mineral content (BMC) between the three groups (Fig. 2C, One-way ANOVA, F_2,23_ = 3.792, P = 0.0377): Compared to CTRL mice, the DIM mice had reduced BMC (Fig. 2C, P = 0.0336) and 8-weeks exercise had a trend of increasing BMC in the DIM-PE group (P = 0.0911). We also analyzed the bone mineral density between the groups (Fig. 2D, One-way ANOVA, F_2,23_ = 3.947, P = 0.0336). We did not find significant difference between the CTRL mice and the DIM mice (Fig. 2D), however, 8-weeks exercise significantly increased BMD (Fig. 2D, P = 0.0258). Though exercise stabilizes the body weight in DIM-PE group, there was no difference in lean mass or fat mass between the DIM-CTRL and the DIM-PE group (Fig. 2E,F).

### Central benefits of PE on cognitive function

**Figure 3:**
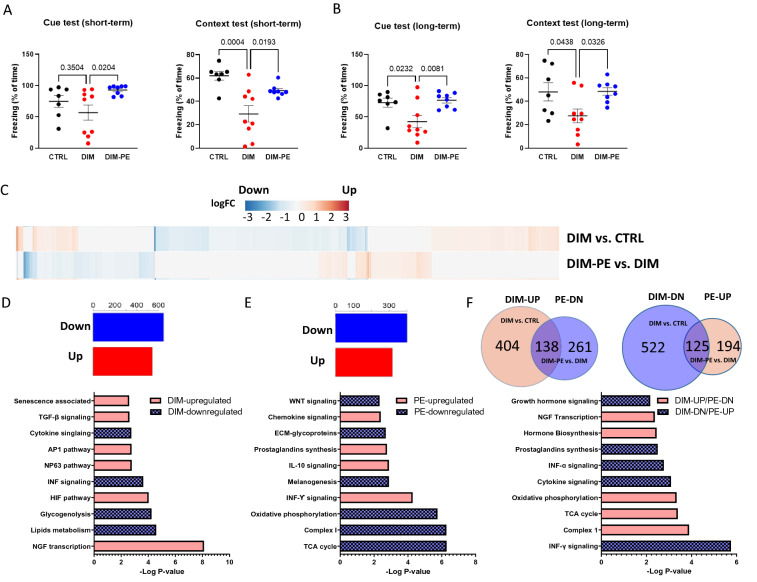
The effect of high fat diet and physical exercise intervention on learning and memory and transcriptional assessment of hippocampus in response to diet and physical exercise. (**A**) Short-term memory assessed by fear conditioning, % of freezing in the cue test and context test. (**B**) Long-term memory assessed by fear conditioning, % of freezing in the cue test and context test (**C**) Heatmap of genes differentially regulated by high fat diet (DIM vs. CTRL) compared with the same genes regulated by physical exercise (DIM-PE vs. DIM). (**D**) Number of upregulated genes and downregulated genes in DIM mice compared to CRTL mice and top 10 GO enrichment pathway analysis of DEGs regulated by high fat diet. (**E**) Number of upregulated genes and downregulated genes in DIM-PE mice compared to DIM mice and top 10 GO enrichment pathway analysis of DEGs regulated by PE intervention. (**F**) Venn diagrams of total number of significantly upregulated and downregulated genes in high fat treated mice with or without PE intervention and top 10 GO enrichment pathway analysis of DEGs regulated by high fat diet and rescued by PE intervention. Data represents mean ± SEM. n = 7–9 per group for (**A**) and (**B**).

To test whether MetS developed during middle age affects cognitive function and whether PE has cognitive benefits, we subjected all mice to a contextual fear conditioning test. We found that the DIM mice had short-term memory deficits compared to the CTRL and the DIM-PE mice reversed the MetS-induced learning and memory deficits (Fig. 3A, One-way ANOVA, F_2,21_ = 3.976, P = 0.0343 for auditory memory and F_2,21_ = 10.52, P = 0.0007 for context memory). Long-term memory test also showed that the DIM mice had impairments in memory consolidation. The DIM mice had significantly decreased freezing in both the context and the auditory memory test (Fig. 3B, One-way ANOVA, F_2,21_ = 6.264, P = 0.0074 for auditory memory and F_2,21_ = 4.442, P = 0.0246 for context memory).

### Gene expression assessment in the hippocampus in response to DIM and to PE

Hippocampal formation plays an important role in learning and memory. To test whether exercise can modulate DIM-induced gene expression changes in the hippocampus that may contribute to memory function, we isolated RNA from the hippocampus and performed RNA-seq. Heatmaps of transcripts that were significantly regulated are shown in Fig. 3C. There were 1189 DIM-induced DEGs (542 DIM-upregulated DEGs and 647 DIM-downregulated DEGs, Fig. 3D). Top GO terms enriched with DIM-induced upregulated genes were related to NGF transcription, HIF pathway, NP 61 and AP1 pathway, TGF-β signaling and senescence associated pathway. DIM-induced downregulated pathways are associated with lipid metabolism, gycogenolysis, interferon (INF) and cytokine signaling (Fig. 3D). Compared to the DIM, there were 718 PE-mediated DEGs (319 PE-upregulated DEGs and 399 PE-downregulated DEGs, Fig. 3E). Top GO terms enriched with PE-induced upregulated genes were mostly related to immune responses (INF-γ, IL-10, chemokine signaling and prostaglandins synthesis) while PE-downregulated genes were mostly related to metabolism (TCA cycle, complex 1, oxidative phosphorylation) and extracellular matrix (ECM)-glycoproteins (Fig. 3E). Among the 542 DEGs that were upregulated in DIM, 138 were rescued by exercise and among the 647 DEGs that were downregulated in DIM, 125 were reversed by exercise (Fig. 3F). Rescue assessment showed that DIM-induced downregulated pathways that were related to immune responses (INF-γ signaling, INF-α signaling, cytokine signaling and prostaglandin synthesis) and growth hormone signaling were reversed by exercise (Fig. 3F). DIM-upregulated pathways that were related to mitochondrial function (TCA cycle, oxidative phosphorylation) and NGF transcription were reversed by exercise (Fig. 3F).

### Effect of PE on peripheral biochemistry

To assess the effect of DIM and PE on metabolic hormones, we collected plasma and conducted ELISA using the mouse hormone multiplex kit. We found that compared to CTRL mice, DIM mice had significantly increased levels of Insulin, C-peptide, a proinsulin that is important for insulin synthesis and pancreatic polypeptide (PP), an endocrine regulates pancreatic secretion activities and impacts liver glycogen storage and gastrointestinal secretion (Table 1). There was also a significant increase of leptin, an important hormone secreted mainly by adipocytes and directly reflects the total amount of fat in the body and glucagon-like peptide -1 (GLP-1), a peptide hormone secreted by intestinal L-cells that regulates insulin secretion (Table 1). We found that 8-weeks PE had no effect on the levels of any of these hormones in circulation albeit improvement in the glucose tolerance test (Table 1).

**Table 1:** Blood biochemistry assessment in control, DIM and DIM-PE mice Plasma levels of metabolic syndrome-associated hormones and cytokines measured at the end of the study. Data represents mean ± STDEV, n = 7–9 per group.

	CTRL	DIM	DIM-PE	P (One-way ANOVA)	P (DIM-PE vs. DIM)
C-peptide	441.7 ± 172.1	1415.7 ± 593.8	1318.9 ± 558.2	0.011	0.9414
Insulin	884.7 ± 257.2	4456.1 ± 4011.2	3575.3 ± 2615.0	0.0432	0.765
PP	103.4 ± 57.7	46.6 ± 21.4	59.0 ± 44.7	0.0328	0.7952
Leptin	6819.4 ± 3539.3	29,320.1 ± 19,948.3	24,788.3 ± 9493.6	0.0048	0.7222
GLP-1	559.0 ± 300.3	1141.0 ± 663.2	1038.7 ± 503.6	0.0003	0.889
GIP	71.1 ± 46.0	343.1 ± 357.4	349.4 ± 281.0	0.0758	0.9984
PYY	132.5 ± 43.3	131.1 ± 46.7	152.7 ± 56.4	0.5932	0.5921
Glucagon	55.3 ± 33.4	70.8 ± 32.0	115.2 ± 107.2	0.6729	1
Amylin	54.6 ± 40.0	62.3 ± 42.1	58.7 ± 40.9	0.8762	0.8505
secretin	54.8 ± 62.0	29.8 ± 16.0	29.8 ± 21.3	0.523	0.765
IL-6	49.1 ± 43.7	210.8 ± 231.0	38.5 ± 33.8	0.042	0.046
TNF-α	8.7 ± 3.4	19.9 ± 9.1	20.2 ± 8.9	0.0086	0.9974
MCP-1	54.3 ± 19.7	84.3 ± 51.5	58.8 ± 33.6	0.261	0.3266
Cholesterol	1.6 ± 0.2	2.6 ± 0.4	2.5 ± 0.5	< 0.0001	0.8755
Triglyceride	1.7 ± 0.7	2.9 ± 0.8	2.0 ± 0.5	0.004	0.0167

Inflammation is closely associated with metabolic syndrome. There was an increase in the levels of IL-6, TNF-α (Table 1) but there was no difference in the level of chemokine monocyte chemoattractant protein-1 (MCP-1) which was shown to play an important role in renal injury and fibrosis in diabetic nephropathy^36^. 8-Weeks PE significantly reduced the plasma level of IL-6, however, had no effect on TNF-α (Table 1). We then measured the level of cholesterol and triglyceride, we found that DIM mice had significantly higher plasma level of total cholesterol and triglyceride and PE significantly lowered the level of triglyceride but not cholesterol (Table 1).

## Discussion

Metabolic syndrome is a multifactorial disease that manifests its pathological features both in the periphery and in the central nervous system. Previous preclinical studies using a high fat diet to induce hyperinsulinemia, hyperglycemia and hyperlipidemia often start the diet at a very young age in order to study the effect of life-long metabolic disturbance. Since the prevalence of MetS increases with age and more middle-aged people are affected by this disease we decided to study the effect of MetS developed during that period of life on periphery and cognitive health.

Physical activity has been recommended to lower the prevalence or risk of MetS^28–30,37–40^. However, epidemiological studies examining the association between physical activity and the risk of MetS reported conflicting findings. Some suggested that PE may benefit metabolic and cardiovascular function and reduce risk factors related to MetS^39–44^. Some showed no benefits^45–48^. The discrepancy is very likely related to the types and intensity of exercises or physical activities involved in the studies. We show that a 5-month western diet led to increased body weight, hyperglycemia, hyperlipidemia and insulin intolerance in middle-aged female mice while 8-week voluntary running wheel exercises stabilize body weight and significantly improve glucose tolerance albeit continuing on the high fat diet. Assessment of plasma levels of molecules that are related to glucose control did not find improvement in the levels of insulin, PP, GLP-1 or glucagon. Previous studies demonstrated that running wheel exercise-mediated improvements in blood glucose is primarily attributed to the reduction in the endogenous glucose production which is largely controlled by insulin and glucogon^49^. It is possible that our assessment of glucose tolerance was conducted immediately after the running wheel exercises while the measurement of these hormones were conducted weeks after the mice completed their exercise. Moreover, most of the studies showing molecular changes that are associated with improved glucose uptake and glucose metabolism were conducted using forced instead of voluntary exercise^50–55^. Forced exercises usually subject mice or rats to motor-driven treadmill and provide a much higher intensity of the physical activity. Generally speaking, the treadmill runs at a rate of 10–25 m per minute and in our voluntary wheel running the mice ran about an average of 4100 m over the 24 h recording period. It is possible that the differences in the intensity of exercise may have different impact on plasma levels of molecules related to glucose control.

The prevalence of age-associated bone loss in women is higher than in men and severe osteoporosis in postmenopausal women is also associated with obesity. Previous report showed that female C57BL/6 mice fed with a 10% corn oil high fat diet led to significant bone loss as reflected by lower bone mass density (BMD)^56^. In our study, we found that 6 months high fat diet led to a significantly lower bone mineral content compared to control lean mice while there is no significant difference in BMD. The lack of BMD changes could be due to the different sources of fats used in the diet. Our high fat diet mainly uses animal fats (lard) while corn oil was used in the previous study. Corn oil has high content of omega-6 fatty acids and very low omega-3/omega-6 ratio (1:46), which is postulated to have a big impact on bone losses during aging while the ratio of omega-3/omega-6 in lard is approximately 1:10. Nevertheless, we found two months of wheel-running was able to increase BMD in mice albeit they were continuously fed on the high fat diet. Our data supports the clinical observation that physical exercise is effective in stimulating osteogenesis and reduces the rate of bone loss. The exercise protocol used in the study is voluntary and mice were only subjected to the running wheel three times a week. Therefore, it can be easily recommended for long-term application in humans.

It is well acknowledged that MetS not only affects the periphery but also has a negative impact on the CNS^57–59^. Numerous epidemiological studies have shown that people with MetS have increased risk of developing age-related dementia^13–16^. In animal models, it has also been demonstrated that risk factors associated with metabolic syndrome such as diabetes and obesity can induce neurological deficits, including reduced hippocampal dendritic spine density, reduced LTP and impaired learning ability^60–63^. Previously we found that mice chronically fed on high fat diet starting at a very young age (2 months of age) had abnormal functional connectivity in the brain^64^ and synaptic maladaptation^32,33^. In this study, we showed that development of MetS in middle age can also lead to impairment in both short-term and long-term memory. RNA-seq in the hippocampal formation identified several pathways that are significantly altered in the brain of female mice developed MetS at middle age. Most notably, the lipid metabolism pathway and glycogenolysis pathway are significantly downregulated in the MetS brain. The brain is one of the organs that has very high content and rich composition of lipids and it is considered to be autonomous in lipid synthesis. Astrocytes are the main cell type that produce lipids which are taken up by neurons for synapse formation. The homeostasis of lipids in the brain is vital to proper brain function. Disturbance of lipid metabolism has been linked to several neuropathological conditions such as Alzheimer’s disease and Parkinson’s disease. Glycogen is important energy reserve in the brain and is found predominantly in astrocytes with small amounts in neurons. Specific neurotransmitters and neuronal signaling can trigger glycogenolysis which in turn breaks glycogen down to glucose-1-phosphate and provides fuel for ATPases to pump K^+^ and Ca^++^ for proper brain function and memory. Previous studies showed that MetS associated hyperglycemia may disrupt normal TCA cycle in the mitochondrial matrix^65^ and disturb oxidative phosphorylation, increase production of reactive oxygen species (ROS), leading to impaired mitochondrial biogenesis^66,67^. Our data suggests that astrocytes are very likely the main cell type in the brain that are affected by these metabolic alterations that may lead to impaired glutamine synthesis, loss of synaptic proteins and eventually synaptic maladaptation and brain dysfunction. Previous studies conducted in young animals with metabolic disturbances showed that physical activity can improve brain function by reducing oxidative stress and lipid peroxidation^68–71^, modulating immune responses and reducing neuroinflammation^68,69,72–75^, and improving insulin signaling and glucose utilization^68,75–79^. Our study also showed that two months of voluntary wheel running significantly improves learning and memory. MetS-mediated DEGs rescued by physical exercise are enriched primarily in pathways associated with mitochondrial function and immune responses. Physical exercise significantly downregulates MetS-induced upregulation of TCA cycle and oxidative phosphorylation that may help restore proper energy metabolism in astrocytes, maintain redox potential and glutamate homeostasis, hence improved brain function. Our RNA-seq data conducted in middle aged mice is largely consistent with the mechanisms elucidated in previous studies^68–79^ that physical exercise may benefit metabolic syndrome-mediated cognitive impairment though modulation of redox regulation, neuroinflammation and energy metabolism.

The prevalence of metabolic syndrome has steadily increased over the years and poses a serious burden on the US healthcare system. Our studies provided preclinical evidence that physical activities, exercised voluntarily and three times a week, provides protection against MetS-induced peripheral and central nervous system dysfunctions. Future study will assess clinical benefits of this kind of exercise regimen in humans.

## Methods

### Animal model and treatment

Female C57BL6/J mice were purchased from Jackson’s laboratory and housed in the centralized animal care facility at the James J. Peters Veteran Affairs Medical Center (VAMC). The C57BL/6 J mouse is used to model features of human MetS as it develops obesity, hyperinsulinemia and hyperglycemia, dyslipidemia and glucose intolerance when fed a high fat diet^31^. Mice received standard chow until they reached 1 year of age. At 12 months of age, mice were randomly grouped into two groups and received the following treatments: Control mice were fed with regular diet (LF-CTRL group, 10 kcal% fat, Research Diets D12450B, n = 8), Diet-induced metabolic syndrome (DIM) mice were fed with a high fat diet (DIM group, 60 kcal% fat, Research Diets, D12492, n = 18). Following 5-months high fat diet treatment, the DIM mice were randomly assigned to the exercise (DIM-PE, n = 9) or sedentary control group (DIM-CTRL, n = 9). Both groups of mice continued to be fed on the high fat diet throughout the study. All animals were maintained on a 12:12-h light/dark cycle with lights on at 07:00 h in a temperature-controlled (20 ± 2 °C) vivarium and all procedures were approved by the VAMC IACUC (Approve number WAN-16–061). All proceudres were conducted in accordance with the ARRIVE guidelines and the Guidelines for Animal Care and Use at the James J. Peters VAMC.

### Intra-peritoneal glucose tolerance test (IGTT)

IGTT was performed as previously reported^33,80^ following 5 months treatment and at the end of the physical exercise. Specifically, mice were given a single dose of intraperitoneal glucose administration (2 g/kg BW) following overnight fasting and postprandial blood was collected from the tail vein periodically over a 2 h period. Blood glucose content was assessed using the Contour blood glucose System (Bayer, IN).

### Physical exercise

At 17 months of age, following 5 months of high fat-diet treatment, the DIM mice were randomly assigned to the exercise (DIM-PE) or sedentary control group (DIM-CTRL). The DIM-PE mice were subjected to voluntary running wheel exercise in the Model 80820S Scurry Activity Wheel Chamber (Campden Instruments Ltd.) three times a week for 8 weeks. Their physical activities were recorded.

### Contextual fear conditioning test

Contextual fear conditioning test was performed as previously described with modification^33^. On Day 1, mice were placed into Context A and allowed to explore for 120 s (baseline) prior to three 30 s tone/shock pairings (30 s 4.0 kHz pure tone co-terminating with a 2 s scrambled 0.6 mA foot-shock). Each tone/shock pairing was separated by 30 s of exploration time, and animals were given 30 s to explore following the final tone/shock pairing (300 s total). Thirty minutes after the training, mice were introduced to Context B, exposed to 4.0 kHz pure tone for 240 s and returned to their home cage. Thirty minutes after the tone test, mice were brought back to Context A and allowed to explore for 240 s without the tone. On day 2, mice were placed into Context A, and allowed to explore for 240 s without the tone. On Day 3, mice were placed into Context B and allowed to explore for 240 s in the constant presence of the 4.0 kHz pure tone. Freezing behavior was recorded remotely and analyzed using Stoelting ANY-MAZE Fear Conditioning Software. Memory for short-term and long-term context (contextual memory) or tone (auditory memory) for each animal were calculated.

### DEXA scan

A small animal dual energy X-ray absorptiometer (DXA, Lunar Pixmus, WI) was used to measure areal bone mineral density (BMD)^81^. Briefly, mice were anesthetized with low ketamine/xylazine and placed on the scanner bed in the prone position, with the limbs and tail stretched away from the body. The scan data was analyzed with PIXImus software (2.10; GE/Lunar) according to manufacturer’s instructions.

### Plasma biochemical indexes

Blood was collected using a heparinized capillary tube and plasma was collected following centrifugation at 2000×g for 15 min. Samples were tested using the following commercially available kits: Amylin, C-peptide, ghrelin, gastric inhibitory polypeptide-1 (GIP-1), glucagon, IL-6, insulin, leptin, MCP-1, pancreatic polypeptide (PP), peptide YY (PYY), resistin, secretin and TNFα were measured using the mouse metabolic hormone magnetic bead multiplex MAP kit from Millipore (Billerica, MA); Cholesterol and triglyceride quantitative kits from Biovision (CA).

### RNA isolation and RNA-seq

Total RNA from the hippocampus was isolated using the RNeasy Mini Kit (Qiagen, Valencia, CA). All RNA preparations were confirmed with integrity numbers > 8.0 by a Bioanalyzer before proceeding to library construction. Library construction (LncRNA library, Ribo-Zero, 4–5 libraries per test condition), quality assessment and Illumina Novaseq 6000 with 150-pb paired-end reads were conducted by Novogene.

### RNA-seq data analysis and bioinformatics

Quality control was carried out on the raw sequence data coming from the sequencer using the FastQC software^82^. This assesses total sequence, reads flagged as low quality, read length, GC content, per base and per tile quality, per sequence quality score, per base content distribution, per sequence GC content, per base N content, sequence length distribution, sequence duplication levels, overrepresented sequence, adaptor content, and Kmer content. Reads of universal low base quality were discarded, and reads with certain low quantity bases were trimmed. Following QC, RNA reads were mapped to mouse reference genome (mm10) with STAR aligner^83^ to obtain gene-level read counts. We retained genes with counts per million reads (cpm) > 1 in at least 4 samples for downstream analysis. Read counts within samples and between samples were then normalized and the differential expression level between groups were examined using the edgeR R-package^84^. Genes with 20% fold change and nominal p < 0.05 were considered statistically significant.

### Gene ontology (GO) analysis

GO analyses were performed on differentially expressed genes (DEGs) from different contrasts by using Fisher’s Exact Test (FET) with Benjamini-Hochberg (BH) correction. The annotated genes in the MSigDB canonical pathways were considered as the background. The enriched GO terms with p value < 0.05 were considered statistically significant.

### Statistical analysis

For behavioral test as well as biochemical analyses comparing control group vs. test group(s), two-tailed student t-test (paired or unpaired), one-way ANOVA or two-way-ANOVA repeated measure followed by Post Hoc Bonferroni multiple comparisons were used. In all studies, outliers (2 SD from the mean) were excluded and the null hypothesis was rejected at the 0.05 level. All statistical analyses were performed using Prism 9 (GraphPad Software, San Diego CA).
